# Supplementary material for: Survival Outcomes and Failure Patterns in Patients with Inoperable Non-Metastatic Pancreatic Cancer Treated with Definitive Radiotherapy
Source: Cancers (Basel). 2023 Apr 9;15(8):2213. doi: 10.3390/cancers15082213 (PMC10136775; doi:10.3390/cancers15082213)
Supplement: Supplementary file 1 [file cancers-15-02213-s001.zip › cancers-2228683-supplementary.pdf]

Supplementary Figure S1

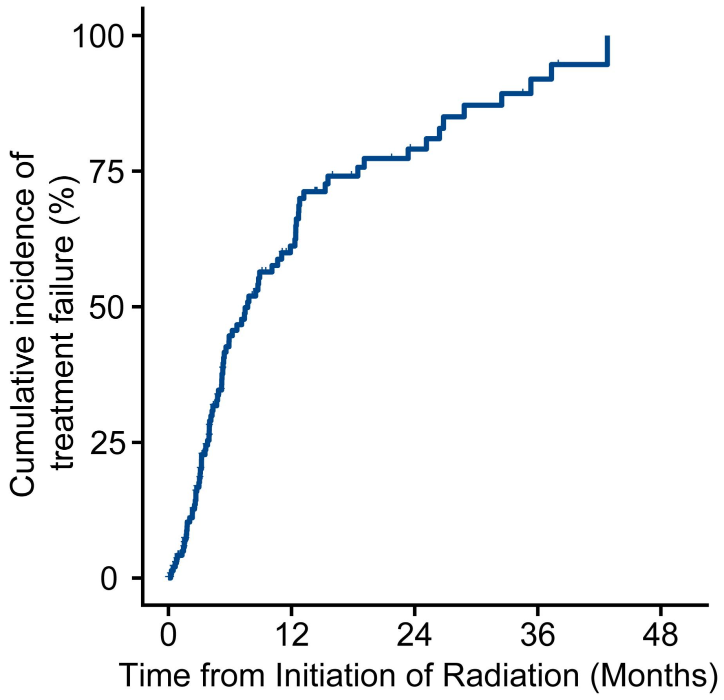

Figure S1: The 1-year and 2-year cumulative incidences of disease progression after RT in entire group.
